# Differentiation of axial spondyloarthritis-related lesions from degenerative lesions on Na[^18^F]Fluoride PET/CT in a longitudinal dataset of patients with radiographic axial spondyloarthritis

**DOI:** 10.1007/s00259-026-08178-6

**Published:** 2026-10-09

**Authors:** Jerney de Jongh, Sam Groothuizen, Gerben J.C. Zwezerijnen, Robert Hemke, Tom Doorschodt, Ralph Paul Maguire, Tom Vaux, Conny J. van der Laken

**Affiliations:** 1 Department of Rheumatology & Clinical Immunology, Amsterdam UMC, Amsterdam, The Netherlands; 2 Department of Radiology & Nuclear Medicine, Amsterdam UMC, Amsterdam, The Netherlands; 3 Department of Radiology & Nuclear Medicine, OLVG, Amsterdam, The Netherlands; 4 UCB Biopharma SRL, Brussels, Belgium; 5 UCB Pharma, Slough, United Kingdom

**Keywords:** Na[^18^F]Fluoride, Positron emission tomography, Radiographic axial spondyloarthritis, Ankylosing spondylitis, Imaging

## Abstract

**Purpose:**

To investigate longitudinal changes of Na[^18^F]Fluoride uptake in the axial skeleton of patients with radiographic axial spondyloarthritis (r-axSpA) during biological treatment, focusing on r-axSpA and degenerative (D) lesions.

**Methods:**

20 active r-axSpA patients were included. Na[^18^F]Fluoride Positron Emission Tomography / Computed Tomography (PET/CT) scans were performed prior to, as well as 12 and 52 weeks after starting biological therapy. PET-positive lesions in the axial skeleton were scored and classified by two blinded readers. Quantitative Na[^18^F]Fluoride uptake was assessed by determination of standardized uptake values (SUV) corrected for individual integrated whole blood activity concentration (SUV^AUC^).

**Results:**

A total of 276 lesions with visual Na[^18^F]Fluoride uptake were detected, of which 98 (36%) were defined as r-axSpA and 46 (17%) as D. The total number of r-axSpA lesions decreased from 98 at baseline to 67 at week 12 (-32%) and to 55 at week 52 (-18%), while the number of D lesions remained stable over time. The majority of r-axSpA lesions were observed in the thoracic spine (84%), while the majority of D lesions were observed in the lumbar spine (48%). Quantitatively, 84% and 88% of the r-axSpA lesions showed a decrease of Na[^18^F]Fluoride uptake between baseline and 12 and 52 weeks, respectively. In contrast, most D lesions either showed an increase or mild decrease between baseline and follow-up.

**Conclusion:**

Na[^18^F]Fluoride uptake in visually classified r-axSpA and D lesions in r-axSpA patients on PET/CT demonstrated a striking different change in both the number of r-axSpA lesions and quantitative tracer uptake herein versus D lesions while on biological treatment.

**Supplementary Information:**

The online version contains supplementary material available at https://doi.org/10.1007/s00259-026-08178-6.

## Introduction

Radiographic axial spondyloarthritis (r-axSpA), previously known as ankylosing spondylitis (AS) is a chronic inflammatory rheumatic disease primarily affecting the axial skeleton, resulting in progressive structural damage and functional impairment [1]. Imaging plays an important role in the objective assessment and monitoring of r-axSpA disease activity [2]. It can be challenging to differentiate between r-axSpA-specific and degenerative lesions, such as osteoarthritis, which are common in the aging population [3, 4]. Moreover, secondary osteoarthritis poses a significant challenge for r-axSpA patients [5]. Chronic inflammation and mechanical stress in r-axSpA can lead to early structural damage, accelerating the development of degenerative lesions [6, 7]. This cumulative damage compromises joint and spine integrity and complicates treatment in this relatively young patient group. Reliable and accurate assessment of r-axSpA disease activity and differentiation from primary or secondary degeneration is crucial for optimal management and treatment decisions in r-axSpA patients.

Traditionally, conventional imaging modalities, such as X-ray and magnetic resonance imaging (MRI), have been used to assess and monitor structural changes and inflammatory activity in r-axSpA [2, 8, 9]. However, these modalities have limitations in distinguishing between r-axSpA-related lesions and degenerative lesions due to overlapping imaging features such as bone proliferation and osseous reaction with bone marrow edema or fatty infiltration. This can make it difficult to attribute these findings to a specific cause. Furthermore, r-axSpA and degenerative changes can coexist, further complicating the interpretation of MR images [10]. Although conventional X-ray can detect structural changes, it typically takes at least two years for these changes to become visible [2, 11]. Recently, Na[^18^F]Fluoride positron emission tomography/computed tomography (PET/CT) imaging has emerged as a promising tool for detection, quantification and monitoring of skeletal disease activity in r-axSpA patients.

Na[^18^F]Fluoride PET/CT uses fluoride’s high affinity for hydroxyapatite crystals to enable visualization of active bone remodeling and increased mineralization, and hereby reflects osteoblastic activity and associated new bone formation in r-axSpA [12, 13]. This technique offers advantages over conventional imaging by providing quantitative measurements of osteoblastic activity at a molecular level, even prior to development of structural damage visible on conventional X-rays. Several studies have demonstrated the potential of Na[^18^F]Fluoride PET/CT in detecting and monitoring osteoblastic activity and associated new bone formation prior to and after treatment in r-axSpA patients [13]. On the other hand, degenerative lesions can also exhibit Na[^18^F]Fluoride accumulation in regions of heightened bone metabolism, indicating ongoing bone remodeling processes [14]. The methodology to visually distinguish between r-axSpA-related and degenerative lesions on Na[^18^F]Fluoride PET/CT in r-axSpA patients has not been fully explored yet. Longitudinal data on lesional Na[^18^F]Fluoride dynamics may additionally be informative about the nature of the lesion. Due to lack of proven efficacy of biologics on degenerative lesions [15], it is to be expected that Na[^18^F]Fluoride uptake will mainly stay stable during such treatment while tracer uptake in r-axSpA suspected lesions is more likely to show changes as has been previously indicated during anti-TNF treatment [13]. Therefore, in the current study, we pre-defined the visual identification and classification methodology of r-axSpA and degenerative lesions in the axial skeleton in clinically active r-axSpA patients and investigated the longitudinal changes of Na[^18^F]Fluoride uptake in these two types of lesions during biological treatment.

## Methods

### Patients

Consecutive r-axSpA patients were included between October 2017 and May 2020. This study was a substudy of the AS0013 trial, a phase 2 A, multicentre, randomized, double-blind, parallel group, exploratory study of bimekizumab (BKZ) (with certolizumab pegol (CZP) as reference drug), which was conducted at 38 sites in 8 countries across Europe, Russia and the United States [16]. The 20 patients in this substudy were all included from European sites. Patients (≥ 18 years) with moderate to severe active r-axSpA, defined as Bath Ankylosing Spondylitis Disease Activity Index (BASDAI) ≥ 4 and spinal pain (BASDAI item 2) ≥ 4 [17], were recruited. All patients fulfilled the modified New York (mNY) criteria, defined by documented radiographic evidence of sacroiliitis (grade ≥ 2 bilateral or grade 3–4 unilateral), ≥ 3 months symptom duration and age of onset < 45 years [18]. Additionally, patients also had high sensitivity C-reactive protein (hs-CRP) levels greater than the upper limit of normal (ULN; defined as 5 mg/L) at screening and had a prior inadequate response, contraindication, or tolerance to ≥ 2 NSAIDs. Current NSAID therapy was permitted if the patient was receiving a stable dose from 2 weeks prior to baseline. Exclusion criteria were the use of > 1 anti-TNF therapy or a primary failure to any anti-TNF therapy (defined as no response within the first 12 weeks of treatment), previous use of BKZ, CZP or biological therapy other than 1 anti-TNF. Patients with total ankylosis of the spine or inflammatory arthritic conditions other than r-axSpA were excluded; prior diagnosis of inflammatory bowel disease (IBD) was permitted provided the patient had no active symptoms at screening and baseline. Other exclusion criteria included active infection (except common cold), history of chronic or recurrent infections, diagnosis of active tuberculosis (TB), high risk of acquiring TB or latent TB, presence of a secondary, noninflammatory condition (e.g. fibromyalgia) with potential to interfere with study evaluations, and significant neuropsychiatric disorder indicated by: lifetime history of suicide attempt, suicidal ideation in the past 6 months (according to questions 4 and 5 of the Columbia-Suicide Severity Rating Scale [CSSRS] at screening), or depression score ≥ 10 or anxiety score ≥ 15 on the Hospital Anxiety and Depression Scale (HADS) at screening.

### Na[^18^F]Fluoride PET-CT scanning and image acquisition

Na[^18^F]Fluoride PET/CT scans of the SIJ and spine were performed at screening (≤ 14 days prior to baseline), week 12 (≤ 14 days after) and week 48–52 (≤ 14 days after). Whole-body PET-CT scan was performed, covering skull base to mid-thigh. An average of 100 MBq Na[^18^F]Fluoride was injected at baseline, week 12 and week 52. Standard low-dose CT scans were performed for attenuation correction and anatomical location of the PET signal. Patients were scanned in supine position, with either their hands placed on their lap or their arms placed above the head.

Patients were imaged dynamically from 0 to 30 min after i.v. injection of Na[^18^F]Fluoride, over the region of the aortic root and/or left ventricle of the heart and thoracic vertebral column (one field of view). The recommended dynamic framing at all sites was frames of 1 × 10, 4 × 5, 2 × 10, 2 × 20, 4 × 30, 4 × 60, 1 × 150 and 4 × 300 s. This time period included the attenuation scan. Following the dynamic scan, patients were able to move around for 15 min and empty their bladder. During the dynamic PET scanning protocol, 3 venous blood samples of 5 ml were withdrawn as validation for quantification of the arterial blood pool on the images. 45 min post injection a whole body scan (caudal to cranial direction) of 5 min per field of view was acquired to ensure that the whole spine and SI joints were in the FOV.

Using previously described methods, the PET data were normalized and corrected for attenuation, decay, and scatter. All scans were reconstructed as 144 × 144 matrices with a 4 × 4 × 4 mm pixel size. 22 frames with increasingly longer frame lengths (1 × 10, 4 × 5, 2 × 10, 2 × 20, 4 × 30, 4 × 60, 1 × 150 and 4 × 300 s) were created from the dynamic scans. For visual analysis, images were transferred to offline workstations.

### Imaging analysis

Two independent readers, a board-certified musculoskeletal radiologist (R.H. or T.D.) and a board-certified nuclear medicine specialist (G.Z.), who were blinded to the clinical data, evaluated the static PET/CT scans for the presence of PET-positive lesions in cervical, thoracic (including costovertebral joints) and lumbar spine as well as in the SI joints. All images were assessed as a set of three timepoints in chronological order independently by the readers.

Assessment of these three images simultaneously (as a triplet of three longitudinal timepoints) enabled confirmation of the presence and location of individual lesions at the same level on all three timepoints, using low dose CT as reference. Lesions were scored dichotomously and were defined as being PET positive in case Na[^18^F]Fluoride uptake was ≥ 1.5 higher than the background (defined as mean of the SUVmax, measured centrally in 3 not affected lumbar vertebrae). In exceptional cases, lesions could be classified as r-axSpA-related (r-axSpA) lesions if Na[^18^F]Fluoride uptake ranged from > 1.0 to < 1.5, provided that the intensity of uptake was significantly higher on one side compared to the locoregional and contralateral vertebral/sacroiliac side without signs of degenerative lesion morphology as described below. Additionally, classification as an r-axSpA lesion could also be assigned if uptake was observed in an anatomical site highly suspected to be associated with r-axSpA (see classification methodology below). The visual analysis was conducted using standard 3D image viewing software (Philips Intellispace), with the low-dose CT scan utilized as an anatomical reference.

Readers classified lesions as being probably r-axSpA or as degenerative (D), based on a combination of the following overarching principles:

1. Location: D lesions typically affect the intervertebral discs and often facet joints (if combined with D morphology as described under 2. below).
2. Morphological characteristics: D lesions on low-dose CT scans commonly manifest as disc degeneration, osteophyte formation, and facet joint/costovertebral joint hypertrophy. These changes are often associated with disc space narrowing, endplate irregularities, and the presence of osteophytes.
3. In r-axSpA, CT findings can show erosions, sclerosis, and joint space widening primarily affecting the sacroiliac joints and the vertebral corners from which syndesmophytes can develop. Syndesmophytes can bridge adjacent vertebral bodies, resulting in a characteristic bamboo spine appearance.

Lesions that did not visually classify as either r-axSpA or D were marked as undifferentiated. R-axSpA and D lesions were selected for inclusion in analysis if there was concordance in the lesion classification between the two readers. Undifferentiated lesions were left out of further analysis. While this reduces the power of the analyses, it also allows greater confidence in lesion classification.

Following visual assessment of the scans, quantitative assessment of tracer uptake was performed by manually drawing volumes of interest (VOIs), using data analysis software that was developed in the Amsterdam University Medical Center [19]. Fixed size VOIs were manually drawn on top of visual PET-positive lesions that were determined as definitive r-axSpA or D (scored positive as r-axSpA or D by both readers) as well as on locations where visual PET-positive lesions appeared or disappeared before or after treatment in order to calculate a delta. Peak Standardized Uptake Values (SUVs) of Na[^18^F]Fluoride corrected for individual integrated whole body activity concentration (SUVauc) were used for quantitative analysis. In order to measure the image derived input function (IDIF), a fixed size VOI of 1.6 mL was manually drawn in the descending aorta.

### Statistical analysis

All statistical analyses were based on a modified PET-PPS analysis set (*n* = 20). This was the PET-PPS analysis set, excluding patients without evaluable baseline PET/CT scans.

Change from baseline values was calculated both for the number of lesion and SUVauc values. Summary statistics were presented based on observed case data. A comparative analysis of change from baseline in SUVauc for r-axSpA lesions with D lesions was performed at the week 12 timepoint. This analysis used a repeated measures mixed model, using all lesion data available from the PET-CT analysis, including data from the week 52 timepoint, to maximize the predictive power. Mean values for each patient and visit combination were taken for SUVauc prior to modelling to control for the high variation in number of lesions evaluated from the patients. The model itself included baseline SUVauc, lesion type, randomized treatment and visit, along with interaction terms for treatment by lesion type and visit by lesion type.

## Results

### Patient demographics and baseline characteristics

The substudy, (*n* = 20) included r-axSpA patients that fulfilled the inclusion criteria and were assessed with Na[^18^F]Fluoride PET/CT at baseline, week 12 and week 52. Patients had either been treated with BKZ (*n* = 13) or with CZP (*n* = 7). As stated in the methods section, data from the two treatment arms were pooled for the purpose of the analysis in this study. The patient demographics and baseline characteristics are summarized in (Table 1). After 48 weeks, 14/20 (70%) patients met the ASAS40 response criteria. Longitudinal clinical data can be found in (Supplementary Table 3).

**Table 1:** Patient demographics and baseline characteristics

mean (SD), unless otherwise stated	All patients *n* = 20
Sex, male, n (%)	16 (80.0)
Age, years	40.5 (10.1)
≥18–<65 years, n (%)	20 (100)
≥65–<85 years, n (%)	0
HLA-B27 positive, n (%)	19 (95)
BMI, kg/m^2^	26.6 (5.9)
Time since diagnosis, years	5.5 (6.8)
Symptom duration, years	12.9 (9.6)
hs-CRP, mg/L, geometric mean (geometric CV, %)	10.7 (277.3)
ASDAS	3.8 (0.8)
ASDAS-HD, n (%)	7 (35.0)
ASDAS-vHD, n (%)	13 (65.0)
BASDAI	6.2 (1.4)
Total spinal pain	7.0 (1.7)
Nocturnal spinal pain	6.8 (1.5)
BASFI	5.8 (1.5)
PtGADA	6.1 (1.6)
Prior TNFi exposure, n (%)	0
History of uveitis, n (%)	2 (10.0)

Abbreviations: *ASDAS* Ankylosing Spondylitis Disease Activity Score, *ASDAS-HD, ASDAS* high disease activity, *ASDAS-vHD, ASDAS *very high disease activity, *BASDAI *Bath Ankylosing Spondylitis Disease Activity Index, *BASFI* Bath Ankylosing Spondylitis Functional Index, *BMI* body mass index, *CV* coefficient of variation, *HLA-B27* human leukocyte antigen B27, *hs-CRP* high-sensitivity C-reactive protein, *n* number of patients, *PtGADA* patient global assessment of disease activity, *SD* standard deviation, *TNFi* tumor necrosis factor inhibitor

### Visual analysis of F-18-Fluoride PET/CT data

**Fig. 1:**
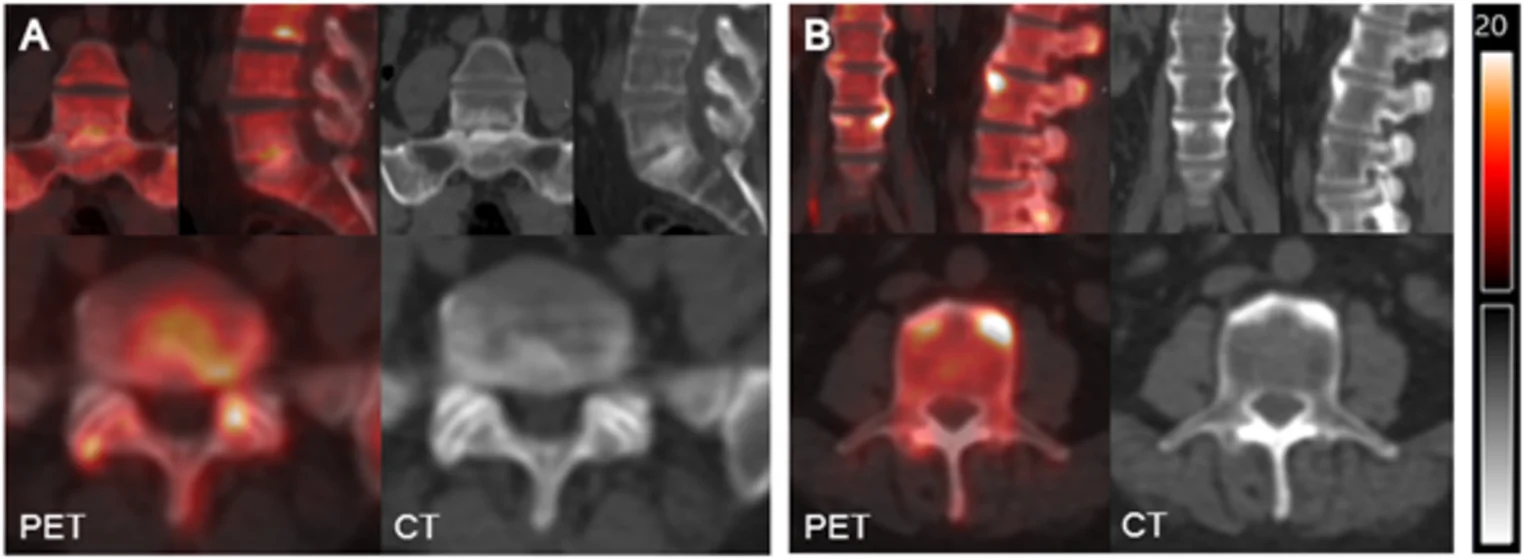
Representative radiographic axial spondyloarthritis (r-axSpA) and degenerative (D) lesions in two different patients on PET/CT scan: (**A**) D lesion at the L5-S1 level. (**B**) r-axSpA lesion at the L2-L3 level demonstrating the formation of a bridging syndesmophyte. Lesions are displayed in the coronal (top left), sagittal (top right), and axial (bottom) planes

In 20 patients, 276 lesions with uptake of Na[^18^F]Fluoride were visually detected by the readers. The number of lesions per patient ranged from 2 to 42. Out of 276 lesions, 98 (35%) lesions were scored as r-axSpA and 46 (17%) as D by both readers. These 144 lesions were used in further analysis. There were no instances where a lesion was classified as r-axSpA by one reader and as D by the other reader. Discordant findings were only found in 61 (22%) of the lesions, when one of the readers scored the lesion as undifferentiated instead of r-axSpA or D. The remaining 71 (26%) lesions were scored as undifferentiated by both readers. All 132 undifferentiated lesions either scored as such by one or both readers were excluded from further analyses. In (Fig. 1), representative r-axSpA and D lesions are demonstrated in Na[^18^F]Fluoride PET/CT scans of included r-axSpA patients.

**Fig. 2:**
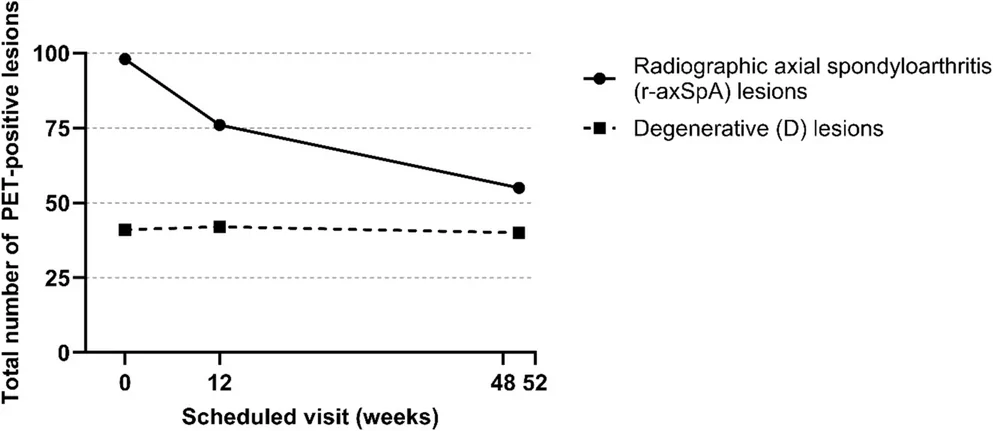
Total number of visually identified and classified r-axSpA (solid line) and D (dashed line) lesions over time

**Fig. 3:**
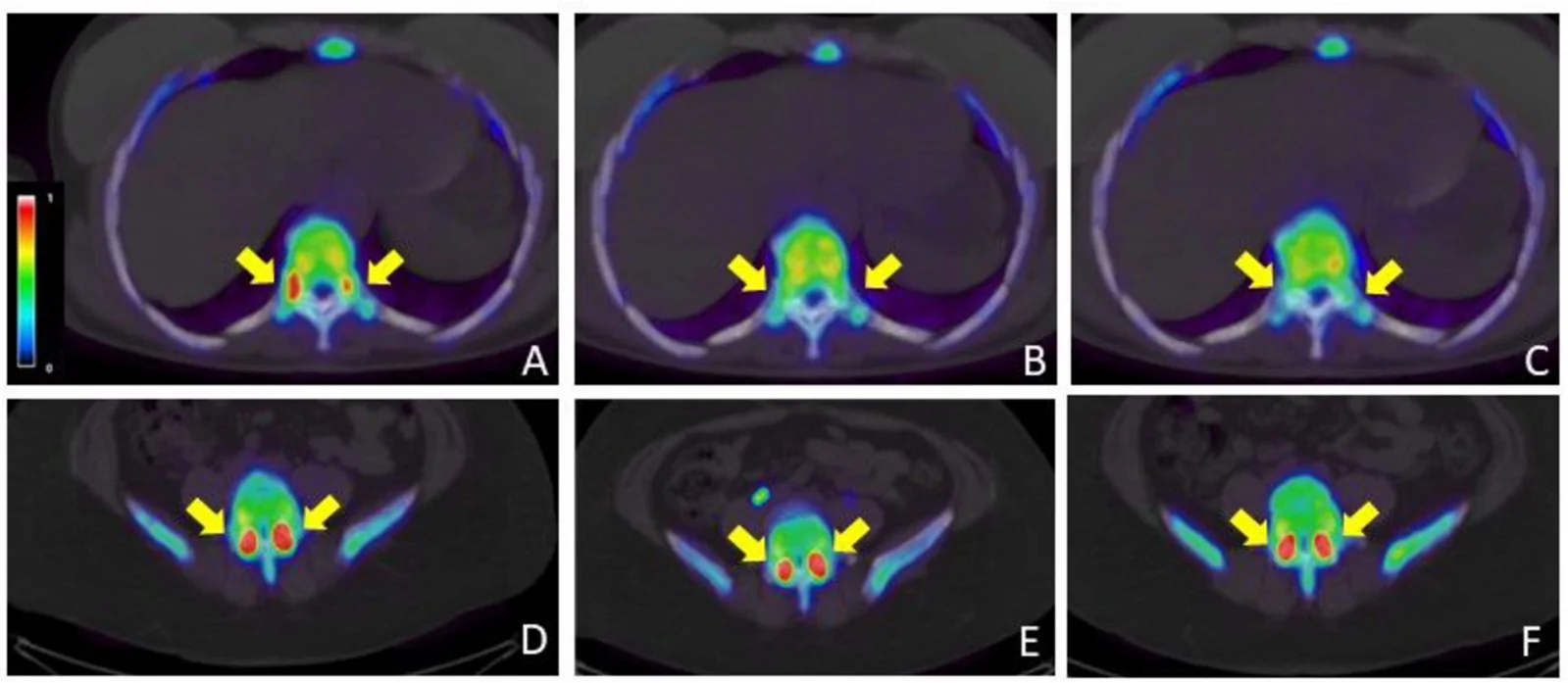
Bilateral r-axSpA lesions at the costovertebral joints in patient 1 at baseline (**A**), week 12 (**B**) and week 52 (**C**), depicting a decrease in Na[^18^F]Fluoride uptake as time progresses. Bilateral D lesions at the L4/L5 facet joints in patient 2 at baseline (**D**), week 12 (**E**) and week 52 (**F**), indicating no significant change in Na [^18^F] Fluoride uptake over the course of the study

The total number of definite r-axSpA lesions decreased from 98 at baseline to 67 after 12 weeks of treatment, which corresponds to a 32% decrease during this interval. Thereafter, the number further decreased to 55 at week 52 (18% decrease after week 12) (Fig. 2; Table 2). In contrast, the number of D lesions remained stable over time, with only a minor change (both increase and decrease) between the range of 40–42 lesions (see Fig. 2; Table 3). Figure 3 illustrates r-axSpA and D lesions on PET/CT scan in two different patients.

Regarding the location of r-axSpA lesions, 74/98 (76%) were found in the spine, with the majority being present in the thoracic region (62/74; 84%), in particular in the thoracic costovertebral joints (37/74; 50%). Almost a quarter 24/98 (24%) of the r-axSpA lesions with Na[^18^F]Fluoride uptake were located in the SI joints. The most pronounced decrease in the number of r-axSpA lesions was detected in the thoracic spine (45% and 12% decrease in 0–12 weeks and 12–52 weeks respectively) including the costovertebral joints, and to a minor extent also in SIJs (29% decrease in 0–52 weeks). At baseline a low number of r-axSpA lesions was detected in the lumbar and cervical spine (Table 2).

In contrast to the observed changes in r-axSpA lesion numbers (in particular decreases in the number of lesions in the thoracic spine), the number of D lesions remained mostly stable in all regions of the spine (Table 3). A more detailed overview of the type of lesions per anatomical region (e.g. facet, endplate of anterior vertebra) is presented in the (Supplementary Tables 1 and 2).

**Table 2:** Distribution of longitudinal changes of total number of radiographic axial spondyloarthritis lesions

	Baseline	Week 12	Week 52
Spine	74	45	38
Cervical	1	1	0
Thoracic Costovertebral	62 37	34 21	30 18
Lumbar	11	10	8
Sacral	0	0	0
SI joint	24	22	17
Total	98	67	55

**Table 3:** Distribution of longitudinal changes in total number of degenerative lesions

	Baseline	Week 12	Week 52
Spine	41	42	40
Cervical	8	8	8
Thoracic	11	12	12
Lumbar	18	18	16
Sacral	4	4	4
SI joint	0	0	0
Total	41	42	40

### Quantitative analysis of F-18-Fluoride PET/CT data

**Fig. 4:**
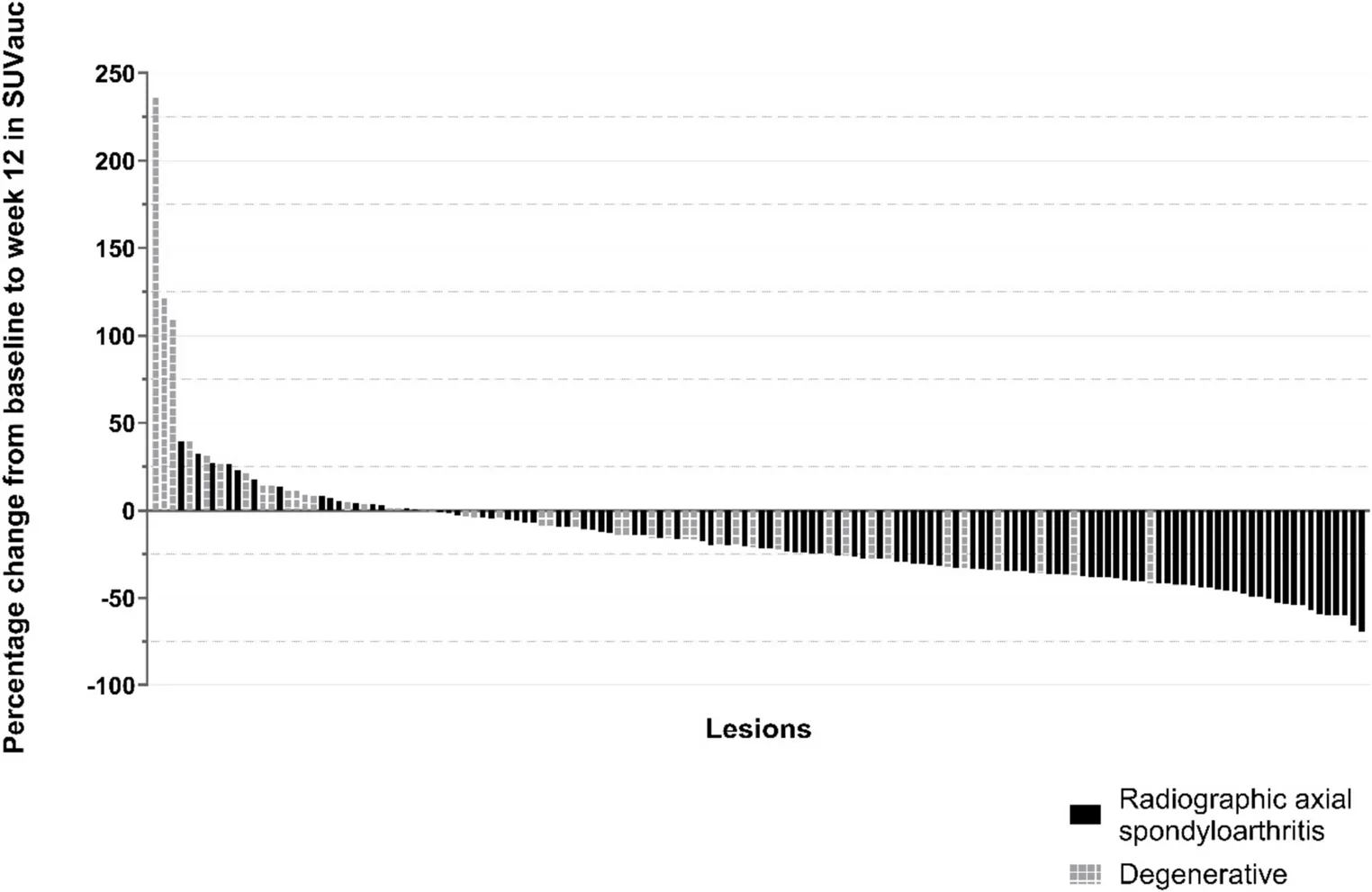
Waterfall plot of percentage change over time (0–12 weeks) in SUVauc of r-axSpA (black) and D (gray) lesions

Quantitatively, 84% and 88% of the r-axSpA lesions showed a decrease in Na[^18^F]Fluoride uptake between baseline and 12 and 52 weeks, respectively. The SUVauc level of Na[^18^F]Fluoride decreased in the majority of r-axSpA lesions with a decrease in SUVauc of up to 69.6% between baseline and 12 weeks. The mean SUVauc per timepoint is shown in (Table 4). The biggest decrease in Na[^18^F]Fluoride uptake occurred in r-axSpA lesions between baseline and 12 weeks (-27.1%). In contrast, most D lesions either showed a large increase (up to > 100%) or a mostly mild decrease between 0 and 12 weeks of treatment (see Fig. 4). Similar patterns were found in the interval of 12–52 weeks.

**Table 4:** Mean SUVauc per timepoint

	Baseline	Week 12	Week 52
r-axSpA	3.76	2.74	2.63
D	3.78	3.53	3.21

Comparative statistical analysis was performed using the repeated measures mixed model as described in the methods section. A highly nominally significant difference between the least squares (LS) mean change from baseline in SUVauc in the r-axSpA lesions compared with the D lesions was observed at week 12 (difference in the LS means) and associated 95% confidence interval (CI) was − 0.84 (-1.446, -0.227) with a nominal p-value of 0.009. This indicates that lesion reduction up to week 12 was significantly greater in the r-axSpA lesions compared with the degenerative lesions. At week 52, a difference was also observed between r-axSpA lesions and degenerative lesions, with a difference in LS means and associated 95% CI of -0.55 (-1.085, -0.015), and a nominal p-value 0.042. This shows a consistently better response in the r-axSpA lesions compared with the D lesions across the duration of the study.

## Discussion

The longitudinal assessments of Na[^18^F]Fluoride uptake in visually classified r-axSpA and D lesions in patients with r-axSpA on PET/CT demonstrated a remarkably different change in both the number of r-axSpA lesions and quantitative tracer uptake herein versus D lesions during biological treatment. The number of r-axSpA lesions decreased between baseline and 1 year with the majority of these lesions showing a quantitative decrease of tracer uptake over time, as compared to the number of D lesions that remained stable over time. These differences were mainly observed in the thoracic spine and to a lesser extent in the SI joints. This is in line with the hypothesis of potential response of r-axSpA lesions to biological treatment and little or no response in D lesions.

Although Na[^18^F]Fluoride uptake has been reported both in r-axSpA and D lesions [13, 20], Na[^18^F]Fluoride PET/CT and PET/MRI studies so far did not report on any sub-analyses of r-axSpA and D lesions, nor on the useful characteristics to distinguish the lesion types [13, 21]. Inclusion of D lesions in the analysis of patients with r-axSpA, may impact Na[^18^F]Fluoride PET/CT/MRI study outcomes, in particular when more established and longer disease duration patients are included. In this study, r-axSpA patients with a long standing disease duration were also included, which resulted in almost 20% of definitely classified D lesions (as assessed by experienced readers). To limit bias, the baseline classification was based on predefined morphological and distribution patterns on PET and low-dose CT. We restricted the analysis to lesions with a high degree of classification confidence and we excluded all undifferentiated or discordant scored lesions. In exploratory checks, the longitudinal behavior of the undifferentiated lesions was broadly distributed across the spectrum of change over time, consistent with a mix of D and r-axSpA-related lesions. They did not show a clear, uniform pattern in one direction, suggesting that their exclusion is unlikely to have introduced a strong systematic bias in favor of either lesion type. The longitudinal observations in our analysis show that the applied visual classification methodology with concordant scoring by the readers performed rather well, at least in thoracic spine and SI joints, assuming that the likelihood of a decrease of Na[^18^F]Fluoride uptake during biological treatment is indeed higher in r-axSpA lesions than in D lesions. Further confirmation of the accuracy of the applied Na[^18^F]Fluoride PET/CT classification of r-axSpA and D lesions could be obtained in future longitudinal studies applying long-term radiological follow-up with typical radiographic r-axSpA and D features as gold standard.

The applied classification in differentiating D and r-axSpA lesions using Na[^18^F]Fluoride PET/CT exhibited notable strengths. Na[^18^F]Fluoride PET/CT provided valuable functional and metabolic information for assessing active bone remodeling, disease progression, and treatment response. The ability to evaluate multiple joint sites simultaneously through whole-body imaging was advantageous for comprehensive assessment. The approach demonstrated potential clinical utility in distinguishing between r-axSpA-related inflammation and degenerative changes in osteoarthritis. Additionally, the comprehensive assessment of lesions using image datasets of the three timepoints simultaneously, with reference to low dose CT, allowed for accurate confirmation and classification based on location. The dichotomous scoring system further ensured standardized lesion identification and characterization, offering clarity and objectivity to the results.

Some limitations should be acknowledged. While Na[^18^F]Fluoride PET/CT provides valuable insights, it does not directly differentiate between the specific underlying pathological mechanisms of osteoarthritis and r-axSpA. The applied classification may have inherent subjectivity, and the accuracy of differentiation relies on the expertise of the interpreting readers. In addition, a ‘gold standard’ for verification is lacking. Although the readers determined the classification of lesions on baseline scans and used triplet scans for verification of the exact location of the lesions over time, they were not blinded for the change in Na[^18^F]Fluoride uptake over time. Hence influence on the classification outcome cannot be ruled out. Despite this known limitation, this was accepted in the applied methodology, since it was considered that the mixing up of lesions at closely located sites could have had more impact on the outcomes of our analysis (based on previously obtained expertise within our group). Moreover, the sample size or patient population in the study may limit the generalizability of the findings. Further validation studies with larger cohorts, including studying known osteoarthritis patients without any rheumatic inflammatory condition, are necessary to establish the reliability and reproducibility of this classification approach. Additionally, considering the availability and current costs of Na[^18^F]Fluoride PET/CT, the practical feasibility and accessibility of this imaging modality in current routine clinical practice warrants consideration. Nevertheless, it is to be expected that accessibility will increase with increased application of PET in a rising number of medical disciplines, which will result in a drop of costs over time. In addition, PET technology is developing rapidly, with new high resolution total body PET scanners currently being introduced into the market. These scanners allow for fast total body scanning with lower radiation burden. In the scope of future developments, the clinical implementation of Na[^18^F]Fluoride PET/CT in r-axSpA patients is poised to undergo significant advancements. One notable area of progress lies in the integration of artificial intelligence (AI) algorithms to enhance the interpretation and analysis of Na[^18^F]Fluoride PET/CT scans. AI-based approaches can aid in automating lesion detection, segmentation and classification, reducing inter-observer variability, and providing more accurate and objective measurements of disease activity and burden. This will also help to better differentiate between disease-specific and degenerative lesions. By leveraging the power of AI, we potentially can obtain more precise and efficient assessments of disease status, leading to improved diagnostic accuracy and treatment decision-making.

Furthermore, the role of Na[^18^F]Fluoride PET/CT compared to MRI in r-axSpA patients is a topic of ongoing exploration. While MRI remains the reference standard for assessing inflammatory activity in r-axSpA, Na[^18^F]Fluoride PET/CT offers complementary information by directly visualizing bone turn-over and osteoblastic activity [22]. Future developments may involve the integration of PET and MRI data [23], utilizing a multimodal imaging approach to provide a comprehensive evaluation of disease activity and its impact on bone health. By combining the strengths of both modalities, a more comprehensive understanding of disease pathology and treatment response can be gained.

Moreover, efforts to establish standardized protocols for Na[^18^F]Fluoride PET/CT imaging in r-axSpA patients should be made. Developing consensus guidelines for acquisition, interpretation, and reporting of Na[^18^F]Fluoride PET/CT scans would promote consistency and facilitate multicenter research collaborations. These guidelines would help ensure the reproducibility and reliability of Na[^18^F]Fluoride PET/CT findings, paving the way for clinical implementation and acceptance.

## Supplementary Information

Below is the link to the electronic supplementary material.

Supplementary Material 1

Supplementary Material 2

## Data Availability

Underlying data from this manuscript may be requested by qualified researchers six months after product approval in the US and/or Europe, or global development is discontinued, and 18 months after trial completion. Investigators may request access to anonymised individual patient–level data and redacted trial documents which may include: analysis–ready datasets, study protocol, annotated case report form, statistical analysis plan, dataset specifications and clinical study report. Prior to use of the data, proposals need to be approved by an independent review panel at www.Vivli.org and a signed data sharing agreement will need to be executed. All documents are available in English only, for a pre–specified time, typically 12 months, on a password protected portal.
