# Supplementary material for: Differentiation of axial spondyloarthritis-related lesions from degenerative lesions on Na[18F]Fluoride PET/CT in a longitudinal dataset of patients with radiographic axial spondyloarthritis
Source: Eur J Nucl Med Mol Imaging. 2026 Oct 9;53(13):7811–9. doi: 10.1007/s00259-026-08178-6 (PMC13727908; doi:10.1007/s00259-026-08178-6)
Supplement: Supplementary file 1 [file 259_2026_8178_MOESM1_ESM.pdf]

Supplementary material

Table 1 – Type of lesion per anatomical region: r-axSpA lesions

r-axSpA	Baseline	Week 12	Week 48/52	Total
**Cervical**	**1**	**1**	**0**	**1**
Vertebra anterior	1	1	0	1
**Lumbar**	**11**	**10**	**8**	**11**
Vertebra anterior	11	10	8	11
**Thoracic**	**62**	**34**	**30**	**62**
Vertebra anterior	5	5	4	5
Costovertebral	18	8	8	18
Costotransverse	37	21	18	37
Facet	2	0	0	2
**Sacroiliac**	**24**	**22**	**17**	**24**

Table 2 – Type of lesion per anatomical region: D lesions

Degenerative	Baseline	Week 12	Week 52	Total
**Cervical**	**8**	**8**	**8**	**8**
Vertebra anterior	1	1	1	1
Endplate	1	1	1	1
Facet	5	5	5	5
Vertebra posterior	1	1	1	1
**Lumbar**	**18**	**18**	**16**	**22**
Endplate	6	6	3	9
Facet	11	11	11	11
Processus spinosus	1	1	2	2
**Sacral**	**4**	**4**	**4**	**4**
Endplate	1	1	1	1
Facet	3	3	3	3
**Thoracic**	**11**	**12**	**12**	**12**
Costovertebral	2	2	2	2
Facet	7	8	8	8
Endplate	2	2	2	2

Table 3 – Clinical follow-up over time

	CZP (n = 7)			BKZ (n = 13)		
	Baseline	Week 12	Week 52	Baseline	Week 12	Week 52
**BASDAI** (mean (SD))	5.6 (0.54)	2.31 (0.86)	1.77 (1.34)	6.58 (1.56)	3.15 (1.70)	3.25 (2.42)
**ASDAS** (mean (SD))	3.36 (0.56)	1.87 (0.58)	1.58 (0.55)	4.02 (0.79)	1.94 (0.80)	2.11 (1.14)
**Total spinal pain** - NRS (mean (SD))	6.43 (1.81)	2.57 (1.81)	1.71 (2.06)	7.23 (1.64)	3.15 (2.58)	3.15 (2.58)
**hs-CRP** - mg/dL (GeoMean (geoCV))	5.48 (1185)	2.33 (273)	1.46 (392)	15.30 (85)	2.05 (186)	1.92 (230)
